# Hybrid Momentum Compensation Control by Using Arms for Bipedal Dynamic Walking

**DOI:** 10.3390/biomimetics8010031

**Published:** 2023-01-12

**Authors:** Zhifa Gao, Xuechao Chen, Zhangguo Yu, Lianqiang Han, Jintao Zhang, Gao Huang

**Affiliations:** 1 School of Mechatronical Engineering, Beijing Institute of Technology, Beijing 100081, China; 2 Faculty of Information Technology, Beijing University of Technology, 100 Pingleyuan, Chaoyang District, Beijing 100124, China

**Keywords:** biped robot, angular momentum, joint torque control

## Abstract

Biped robots swing their legs alternately to achieve highly dynamic walking, which is the basic ability required for them to perform tasks. However, swinging of the swinging leg in the air will disturb the interaction between the supporting leg and the ground and affect the upper body’s balance during dynamic walking. To allow the robot to use its own intrinsic motion characteristics to maintain stable movement like a human when its lower limbs are affected by unknown disturbances during dynamic walking, the ability to use its arms to resist disturbances is essential. This article presents a hybrid momentum compensation control method for torque-controlled biped robots to adapt to unknown disturbances during dynamic walking. First, a hybrid angular momentum and linear momentum regulator is designed to compensate for the disturbance caused by the swinging leg. Second, based on real-time dynamic state changes of the legs, a mixed-momentum quadratic programming controller is designed to realize stable dynamic walking. The proposed method allows the force-controlled robot to maintain its balance while walking down an unknown platform, and it maintains good straightness in the forward direction of dynamic motion. The proposed method’s effectiveness is verified experimentally on the BHR-B2 force-controlled biped robot platform.

## 1. Introduction

Since the initial development of biped robots, researchers have commonly referred to the physiological and physical characteristics of organisms, particularly those of human beings, to propose a series of design methods, balance strategies, movement modes, and control schemes to make the robot safe and stable, and to allow it move naturally within its natural environment like a real organism. Based on the development of the lower limbs, remarkable results have been achieved in terms of controlling the stable motion of the entire body and maintaining the balance of the robot during disturbances by either indirectly or directly controlling the different states of its legs. However, for a long time, little attention has been paid to the important role played by the upper limbs in the movement of biped robots. Because of the nonlinearity of biped robot dynamics, particularly when the robot is subject to a disturbance, the arms have rarely been used to participate in balance recovery or have even been ignored to simplify the generation of the required locomotion patterns [1].

Among the many disturbances that may occur for a biped robot, the disturbance caused by the swinging of its swinging legs is inherent. When the swinging leg swings, a yaw moment will initially be generated around the supporting foot. When this moment exceeds the moment generated by the friction between the supporting foot and the ground, yaw rotation will occur, and the robot will deviate from its predetermined path [2,3,4]. During straight-line walking, the human arm contributes more to the adjustment of the angular momentum of the entire body than the other parts of the body [5], and the arm and the leg extend and retract alternately in the sagittal plane, thus, effectively reducing the rotation of the human body [6]. The arm’s angular momentum compensates for the yaw moment during bipedal walking, which allows the robot to maintain a good straight route [7,8]. In [7], the vertical angular momentum of the biped walking robot is reduced by arm movement in the inverse kinematics task space, especially in the case of a large number of steps. In [8], the yaw moment compensation method based on the swing arm is used to offset the yaw moment caused by the movement of the biped robot while ensuring the z-axis moment balance. Using a similar approach, yaw moment compensation based on multi-objective motion optimization [2] further optimizes the swing arm movement to make it appear more natural and compensate fully for the undesired yaw moment generated by the swinging leg. In addition, by designing a robot mechanism that conforms to the configuration of a human body and its mass and inertia distribution laws, the angular momentum generated during the running process of the robot by controlling the swing of the torso and the arms compensates for the angular momentum generated by the lower body during the flight phase to prevent rotation in the yaw direction [9].

Another disturbance produced during swinging of the swinging leg occurs when the contact between the swinging leg and the unknown terrain changes abruptly; in this case, the upper body will show a tendency to overturn, which disturbs the balance of the robot. In some studies, waist (or pelvis) rotation [10,11] and pelvis combination with arm swinging [12,13] were used to provide momentum compensation by reducing the contact torque between the supporting feet and the ground around the robot’s perpendicular axis during walking, and improving both the straightness of the fast walking trajectory and the upper body’s stability. In [14], a compromise was reached between two strategies for the waist (pelvis) rotation and arm swinging to make the center of gravity more effective for use in tracking the reference trajectory, thus, enabling improved walking stability and efficiency in response to different situations, including model errors and external force disturbances. On the one hand, pelvic rotation makes the nonlinear dynamics of biped robots more complex and also increases the difficulty of motion pattern generation [1]. On the other hand, such a scheme cannot be directly used for many robots without pelvic rotation joints. In addition, the contribution of arm movements to the regulation of human balance increases as the difficulty of the task increases [15,16]. In [17], the mechanism for use of the arm strategy was proposed, and disturbed equilibrium recovery control of a simplified model with arms was realized based on nonlinear model predictive control. The angular momentum generated by swinging of the arms to counteract large disturbances in the upper body and restore overall balance had a positive effect [18,19,20]. In addition, the swinging of the arms plays an important role in maintaining balance when trampling through and colliding with irregular terrain, which is the first problem faced by robots in human environments, but the research in this area has been limited [21].

Although the arm angular momentum is used in the scheme above to compensate for the disturbance caused by the biped robot’s swinging legs, improve the yaw straightness of the robot, and enhance its balance recovery capability, the following deficiencies remain. First, most of the methods above are applied to position-controlled biped robots, where the arm angular momentum compensation mechanism is based on a relatively static reference trajectory, and sudden unknown disturbances to the lower limbs cannot be compensated in a timely manner. For robots with fast dynamic responses and high dynamic walking moment control, including Cassie [22], Digit [23], and Mercury [24], it is necessary to respond to such disturbances in time. Although Cassie and Mercury do not have complete upper bodies that include arms, the role of the arms cannot be ignored, as illustrated in the TORO [19] biped robot. Second, most of these methods only use the angular momentum of the arm swinging while ignoring the real linear momentum, and, thus, do not account fully for the role of the arm.

**Figure 1:**
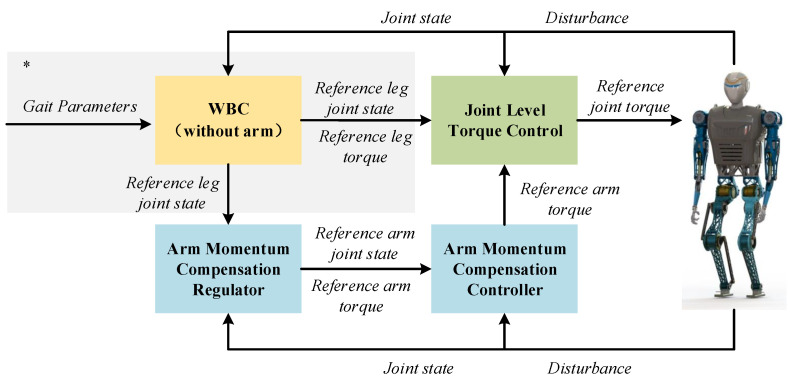
Framework for momentum compensation control of swinging arm. The gray area marked by * is the walking control without arm swing in our previous work [25].

We, therefore, propose a hybrid arm with linear momentum and angular momentum to compensate for unknown disturbances caused by the swinging leg of a biped robot during dynamic walking to reduce the yaw and improve the dynamic stability of this biped robot during dynamic walking. At the same time, based on the motion state of the lower limbs of this biped robot, we use an arm momentum compensation quadratic programming (QP) controller to cause the robot to adjust its momentum in time. The proposed method is validated experimentally using our force-controlled biped robot, BHR-B2. The overall framework is shown in Figure 1. The main contributions of this article are given as follows:

- We propose a hybrid compensation method for both linear and angular momentum by swinging the arms of a force-controlled biped robot to make the robot walk stably, accurately, and dynamically.
- We design a momentum-compensate QP controller based on dynamic adjustment of the robot’s lower limbs that allows the robot to make balance adjustments in a timely manner.

The remainder of this article is organized as follows. Section 2 describes the model of the swinging arm of a biped robot and the momentum compensation method. Next, we construct a dynamic model of the biped robot and design the QP controller in Section 3. Section 4 presents the experiment performed on BHR-B2 to assess the proposed method. Section 5 summarizes our results.

## 2. Swinging Arm Momentum

### 2.1. Swinging Arm Model

**Figure 2:**
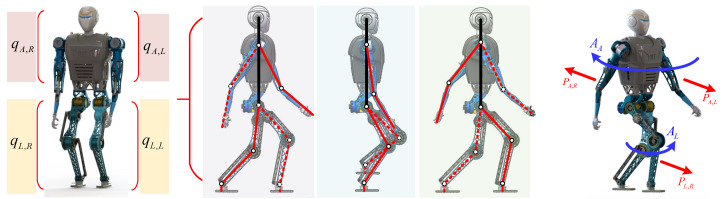
Simplified model of a complete walking cycle for a bipedal robot with swinging arms. The three diagrams in the middle are in the sagittal plane. The solid line in the lower limb indicates the supporting leg, and the dashed line indicates the swinging leg. The solid line in the upper limb indicates the swinging arm corresponding to the supporting leg, and the dashed line indicates the swinging arm corresponding to the swinging leg. The same line type in the upper and lower limbs indicates that they are on the same side of the body. The diagram on the right shows the angular momentum *A* and linear momentum *P* generated by the robot during dynamic walking.

During the movement of a biped robot, similar to step-swing behavior, the arm swings passively in the sagittal plane because of its inertial characteristics. Additionally, the arm and the leg on the same side of the body swing in opposite directions, while the arm and the leg swing in the same direction when located on different sides of the body. Figure 2, shows the swinging process of the arm during a complete walking cycle. The arm’s swing amplitude is proportional to the stride length of the step, the walking speed, the pitching angle of the upper body, and the disturbance that occurs in the direction of travel. In particular, when the robot’s body is disturbed suddenly, the arm’s swing will change greatly in a corresponding manner. The robot’s limb state is denoted by $q=\left\{q_{L,L},q_{L,R},q_{A,L},q_{A,L}\right\}$, wherein the first subscript, *L* represents the leg, and *A* refers to the arm, and in the second subscript, *L* indicates the left and *R* indicates the right.

### 2.2. Arm Momentum Compensation Regulator

When the leg is swinging, the supporting leg is subject to a large rotational moment at the support point. When the resulting torque is greater than the friction torque between the supporting foot and the contact surface, the robot will then produce an unexpected rotational motion that is potentially dangerous. In addition, when the swinging leg contacts the ground during the stepping process, it may result in sudden changes in the lower limbs and even in the whole body state, meaning that the robot is prone to overturning. We refer to the two hazards above as the disturbances caused by the swinging leg during the process of swinging.

We use the angular momentum generated by the swinging of the arm to compensate for the instability caused by the swinging of the leg and, thus, for the potential danger. In addition, we believe that the robot will exhibit passive swinging behavior because of the inertial characteristics of the arm during the process of moving forward, and, thus, we will compensate for the linear momentum of the swinging leg using the swinging arm. We use momentum compensation to ensure that the overall momentum remains in equilibrium, and, thus,

(1) $$A_{A,L}+A_{A,R}+A_{L,L/R}=A_{ref}$$

(2) $$P_{A,L}+P_{A,R}+P_{L,R/L}=P_{ref}$$

where *A* and *P* represent the angular momentum and the linear momentum, respectively. The symbol $\left(/\right)$ represents or and the right subscript ref indicates a reference value. The angular momentum of the arm and the leg depends on the momentum values in the relative directions, i.e., the angular momentum of the left arm and the right leg constitutes a conservation relationship. The linear momentum is the value of the arm and leg moving in the same direction, i.e., the linear momentum values of the left arm and the left leg also form a conservation relationship. Usually, we assume that $A_{ref}=0$ and $P_{ref}=0$. However, it should be noted that to allow the momentum compensation of the arm to be amplified here, we discard the linear momentum of the supporting leg in momentum conservation, and, thus, we have $P_{ref}\neq 0$ in this case. We associate $P_{ref}$ with the arm ${\dot{q}}_{A}$ such that

(3) $$P_{ref}=\eta {\dot{q}}_{A}$$

where η is an empirical coefficient obtained experimentally.

Using Equations (1)–(3), we can obtain the corresponding swinging arm shoulder pitch states ${\dot{q}}_{A,L/R,p}^{{}^{\prime }}$ and ${\dot{q}}_{A,L/R,p}^{{}^{\prime \prime }}$. We select the right arm states ${\dot{q}}_{A,R,p}^{{}^{\prime }}$ and ${\dot{q}}_{A,R,p}^{{}^{\prime \prime }}$ as examples, where

(4) $${\dot{q}}_{A,R,p}^{{}^{\prime }}=I_{A,R}^{-1}\left(A_{ref}-\sum_{i=1}^{3}\left(c_{L,L}^{\left(i\right)}\times P_{L,L}^{\left(i\right)}+I_{L,L}^{\left(i\right)}{\dot{q}}_{L,L}^{\left(i\right)}\right)-2c_{A,R}\times P_{A,R}\right)$$

(5) $${\dot{q}}_{A,R,p}^{{}^{\prime \prime }}=\frac{1}{\eta }P_{ref}$$

where $I_{A,R}^{-1}$ is the inverse of the inertia matrix of the entire arm, and *c* represents the position vector of each part of the arm. In addition, the first subscript of each symbol represents the body part represented by this link to the robot’s torso, for example, *L* is the leg and *R* is the arm; the second subscript of *L* indicates the left and the second subscript of *R* indicates the right, and the third subscript represents the joint axis, for example, *p* represents the joint pitch. To simplify the calculation, we fixed the elbow joint and only considered the role of shoulder pitch $q_{A,R,p}$ in compensation.

However, the coupling mechanism between the supporting leg and the upper body under the combined influence of the angular and linear momentums of the swinging leg remains unclear. Therefore, we distributed the compensation results for this interaction proportionately.

(6) $${\dot{q}}_{A,L,p}=\zeta_{a}\cdot {\dot{q}}_{A,L,p}^{{}^{\prime }}+\zeta_{l}\cdot {\dot{q}}_{A,L,p}^{{}^{\prime \prime }}$$

(7) $${\dot{q}}_{A,R,p}=\zeta_{a}\cdot {\dot{q}}_{A,R,p}^{{}^{\prime }}+\zeta_{l}\cdot {\dot{q}}_{A,R,p}^{{}^{\prime \prime }}$$

where $\zeta_{a}$ and $\zeta_{l}$ are the proportional coefficients for the corresponding angular and linear momentum compensation values, respectively. At the same time,

(8) $$\zeta_{a}+\zeta_{l}=1$$

Then, because of the symmetry of the left and right swinging arms,

(9) $${\dot{q}}_{A,L,p}+{\dot{q}}_{A,R,p}=0$$

Therefore, we integrate Equations (6) and (7) to obtain the left and right arm swings $q_{A,L,p}$ and $q_{A,R,p}$, respectively. The robot’s hardware intrinsically determines the kinematic constraint, where the swing amplitude $q_{A}$ of the arm and shoulder joint and the swing angular velocity ${\dot{q}}_{A}$ must meet the following criteria:

(10) $$\left\{\begin{array}{c} q_{A}^{min}\leq q_{A}\leq q_{A}^{max}\\ {\dot{q}}_{A}^{min}\leq {\dot{q}}_{A}\leq {\dot{q}}_{A}^{max} \end{array}\right.$$

where the superscript min on $q_{A}$ and ${\dot{q}}_{A}$ represents the minimum value in each case, and the corresponding superscript max represents the maximum value in each case.

## 3. Momentum-Compensate Quadratic Programming Controller

### 3.1. Dynamic Model of Robot

The BHR-B2 biped robot has 20 degrees of freedom (DoFs), of which the floating base has six DoFs, and the limbs have 14 driving DoFs, as follows:

(11) $$\left\{\begin{array}{c} q_{f}={\left[x,y,z,\alpha ,\beta ,\gamma \right]}^{T}\\ q_{A,L}={\left[q_{A,L,r}^{\left(s\right)},q_{A,L,p}^{\left(s\right)},q_{A,L,p}^{\left(e\right)}\right]}^{T}\\ q_{A,R}={\left[q_{A,R,r}^{\left(s\right)},q_{A,R,p}^{\left(s\right)},q_{A,R,p}^{\left(e\right)}\right]}^{T}\\ q_{L,L}={\left[q_{L,L,r}^{\left(h\right)},q_{L,L,p}^{\left(h\right)},q_{L,L,p}^{\left(k\right)},q_{L,L,p}^{\left(a\right)}\right]}^{T}\\ q_{L,R}={\left[q_{L,R,r}^{\left(h\right)},q_{L,R,p}^{\left(h\right)},q_{L,R,p}^{\left(k\right)},q_{L,R,p}^{\left(a\right)}\right]}^{T} \end{array}\right.$$

where the superscript on each symbol represents the relevant joint, for example, *s* for the shoulder, *e* for the elbow, *h* for the hip, *k* for the knee, and *a* for the ankle. $q_{f}$ represents the position and attitude of the floating base. In general, the disturbance mainly has a major impact on the sagittal plane motion and state, and the arm swing shows no obvious compensation for the momentum of the roll. Therefore, we ignore the shoulder joint roll DoFs $q_{A,L,r}^{\left(s\right)}$ and $q_{A,R,r}^{\left(s\right)}$ in this case. Because the elbow is fixed, we also ignore $q_{A,L,p}^{\left(e\right)}$ and $q_{A,R,p}^{\left(e\right)}$.

The configuration state of the robot is then given by

(12) $$q={\left[q_{f},q_{A,L},q_{A,R},q_{L,L},q_{L,R}\right]}^{T}$$

The dynamic equation for a biped robot during walking is obtained by using the Lagrange equation as follows:

(13) $$H\left(q\right)\ddot{q}+C\left(q,\dot{q}\right)\dot{q}+G\left(q\right)=S\tau +J^{T}F_{ext}$$

where $H\left(q\right)\in R^{16\times 16}$ is the inertia matrix, $C\left(q,\dot{q}\right)\in R^{16\times 1}$ is the Coriolis force and centrifugal force vector, and $G\left(q\right)\in R^{16\times 1}$ is the gravity vector. $S\in R^{16\times 10}$ is the selection matrix for the driving torque. $\tau \in R^{10\times 1}$ is the driving torque of the leg and arm joints. $F_{ext}\in R^{6\times 1}$ is the external terminal reaction wrench and $J\in R^{6\times 16}$ is the corresponding contact Jacobian matrix. Because we are not considering the force on the contact between the end of the arm and the outside world, the external force acting on the part related to the arm in $F_{ext}$ is zero.

### 3.2. Reference Trajectory

The swing angular velocity and the swing amplitude of each arm are both closely related to the corresponding opposite leg. The swinging amplitude of the arm will increase as the step length of the swinging leg elongates, and the swinging angular velocity of the arm will also increase as the angular velocity of the swinging hip joint of the swinging leg increases. Therefore, the arm joint generates a reference trajectory through a proportional-derivative (PD) controller, as follows:

(14) $${\ddot{q}}_{A}^{d(s)}=k_{P_{1}}\left(q_{A}^{s}-q_{A}^{s}\right)+k_{D_{1}}\left({\dot{q}}_{A}^{s}-{\dot{q}}_{A}^{s}\right)$$

where $k_{P_{1}}$ and $k_{D_{1}}$ are the proportional and differential coefficients of the corresponding PD controller, respectively.

We use the heuristic gait template proposed in previous work to plan the bipedal movements [25]. The heuristic gait template is a model-free method. In this method, the walking process is divided into a support stage and a step stage. The step stage is then divided into the leg lift and step down processes. The end positions and the velocity trajectories of the support leg and the swing leg in the xyz directions were obtained via a unified mathematical template interpolation.

### 3.3. Control

In our previous work [25], the gait template generated the trajectory online and realized motion tracking control through a whole-body dynamic control (WBC) approach. For dynamic motion control of biped robots, please refer to our previous work. In this work, we extend the swing arm momentum compensation control approach based on previous research.

We designed a QP controller to realize momentum compensation of the arm swinging that occurs during walking. The optimal variables for the QP controller are the angular acceleration $\ddot{q}$ and the driving torque τ. According to the different optimization requirements, we divide the objective function into four parts designated $\Omega_{1}$, $\Omega_{2}$, $\Omega_{3}$ and $\Omega_{4}$.

#### 3.3.1. Objective Function

We expect the arm swing to be directly proportional to the swing acceleration ${\ddot{p}}_{sl}^{d}$ of the swinging leg, which is in line with the biological characteristics. Therefore,

(15) $$\Omega_{1}={∥{\dot{J}}_{a}^{\nu }{\dot{q}}_{A}+J_{a}^{\nu }{\ddot{q}}_{A}-\zeta {\ddot{p}}_{sl}^{d}∥}_{\varepsilon_{1}}^{2}$$

where $J_{a}^{\nu }$ represents the linear velocity component of the arm Jacobian. ζ is the proportionality coefficient, which was obtained based on experimental experience. ${\ddot{p}}_{sl}^{d}$ is the desired acceleration at the end of the swinging leg, which is calculated using the PD controller from the position of and the velocity at the end of the swinging leg. In addition, ${∥\cdot ∥}^{2}$ represents the two norms of a vector, and $\varepsilon_{1}$ is the weight matrix of the corresponding objective function term.

The swinging of the arm compensates for the swing momentum of the swinging leg, and the final compensation momentum is expected to be close to the generated momentum, where

(16) $$\Omega_{2}={∥A_{A}+P_{A}+S_{L}A_{L,R/L}+S_{L}P_{L,L/R}-A_{ref}-P_{ref}∥}_{\varepsilon_{2}}^{2}$$

where $S_{L}$ is the swinging leg selection matrix. The angular momentums $A_{L,R/L}$ and momentums $P_{L,L/R}$ are both functions of the expectation ${\dot{q}}_{L}^{d}$ of the swinging leg. $\varepsilon_{2}$ is the weight matrix.

Additionally, the arm joint ${\ddot{q}}_{A}$ must track the results obtained via arm momentum compensation, and, thus,

(17) $$\Omega_{3}={∥{\ddot{q}}_{A}-{\ddot{q}}_{A}^{\left(d\right)}∥}_{\varepsilon_{3}}^{2}$$

where $\varepsilon_{3}$ is the weight matrix and ${\ddot{q}}_{A}^{\left(d\right)}$ is obtained by the PD controller (13) of the arm joint.

In addition, to avoid the instability caused by a sudden change in the joint torque, especially as a result of high-frequency oscillation,

(18) $$\Omega_{4}={∥\tau -\tau^{last}∥}_{\varepsilon_{4}}^{2}$$

where $\varepsilon_{4}$ is the weight matrix. τ and $\tau^{last}$ are the current and last joint torques, respectively.

#### 3.3.2. Constraint

Although the arm swing is doing its best to compensate for the instability and imbalance caused by the swinging leg, the prerequisite is that the constraint conditions are met. Therefore, the hardware constraints, the motion abilities, and the dynamic characteristics of the robot will weaken the momentum compensation effect of the arm swing to some extent.

However, the hardware constraint given by Equation (10) has been constrained in the momentum compensation regulator. In addition, the robot dynamics constraint given by Equation (12) must also be satisfied. Because of the joint motor torque limitation, the torque should be within the following allowable range:

(19) $$\left|\tau \right|\leq \tau_{A}^{max}$$

where $\tau_{A}^{max}$ is the maximum arm joint torque.

#### 3.3.3. QP Controller

Under the condition that constraints (13) and (19) are satisfied, the objective functions (15)–(18) are constructed based on the expectation that the arm swing acceleration will be in step with the swinging leg. The compensation momentum and the generated momentum will cancel each other out, and the torque will not change abruptly. Therefore, the QP controller is designed as follows:

(20) $$\begin{array}{cc} \min_{\dot{q},\tau }\;\;\Omega_{1}+\Omega_{2}+\Omega_{3}+\Omega_{4}\;\;\;\;\;\;\;\;\;\;\;\; & \\ s.t\;\;\;Constraint\;of\;dynamics\;(13)\; & \\ \;\;\;\;\;\;\;\;\;\;\;Joint\;moment\;constraint\;(19)\; & \\ \;\;\;\;\;\;\;\;\;\;\; \end{array}$$

The BHR-B2 robot uses a joint torque control strategy, and each arm joint must be a closed loop for the position and speed of the joint end. Therefore, the final execution moment τ is:

(21) $$\tau_{A,L/R}^{\left(s\right)}=\tau_{A,L/R}+k_{P_{2}}\left(q_{A,L/R}^{d(s)}-q_{A,L/R}^{\left(s\right)}\right)+k_{D_{2}}\left({\dot{q}}_{A,L/R}^{d(s)}-{\dot{q}}_{A,L/R}^{\left(s\right)}\right)$$

where $q_{A,L/R}^{d(s)}$ and ${\dot{q}}_{A,L/R}^{d(s)}$ are the desired states of the shoulder joint, and $k_{P_{2}}$ and $k_{D_{2}}$ are the PD controller coefficients.

## 4. Experimental

Two experiments were conducted on BHR-B2 to verify the effectiveness of the arm momentum compensation control approach. First, experiments with and without arm swing were conducted to verify the positive effects of arm compensation for the disturbance caused by the swinging leg on the contact between the supporting foot and the ground. Second, we subjected the robot to a large disturbance during the walking process to verify the positive effect of arm momentum compensation on the robot’s balance.

### 4.1. Experimental Conditions

#### 4.1.1. Platform

**Figure 3:**
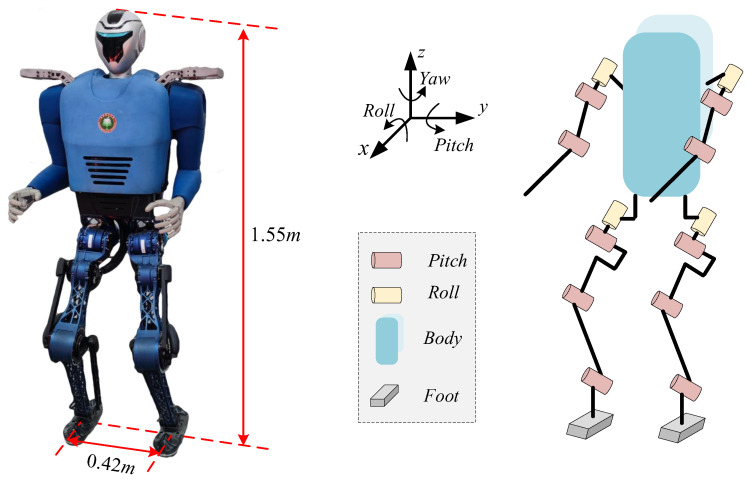
BHR-B2 biped robot platform and configuration of the degrees of freedom.

The BHR-B2 robot is 1.55 m tall, weighs 40.3 kg, and has 14 DoFs, as illustrated in Figure 3 and Table 1. Each leg has four DoFs, comprising the pitch of the hip, the roll of the hip, the pitch of the knee, and the pitch of the ankle. Each arm has three DoFs, comprising the pitch of the shoulder, the roll of the shoulder, and the pitch of the elbow. Each joint is driven using an integrated joint that consists of a brushless DC motor, a planetary reducer with a gear ratio of 17.43, and an encoder.

To make the mass distribution over the whole body more reasonable while also reducing the inertia of the leg, the knee joint drive motor was moved up to the hip, and the ankle joint drive motor was moved up to the knee. After each motor was moved upward, the original position joint shaft was connected through a connecting rod. The design and configuration described above are convenient for achieving precise and direct torque control. A six-axis force/torque sensor is installed between each foot and its ankle to detect each foot’s contact with the ground. An inertial measurement unit (IMU) is installed on the waist of the robot between its hips to measure the actual state of the robot.

#### 4.1.2. Parameter Settings

BHR-B2 uses the EtherCAT communication mode to make the control period up to 0.001 s. The initial step period is set at T=0.4 s, and the maximum forward speed $v_{x}=0.7$ m/s. The arm swing angle range is $-60{}^{\circ}\leq q_{A}^{\left(s\right)}\leq 60{}^{\circ}$. All other parameters are set in Table 2.

In addition, we use Eigen-QuadProg https://github.com/jrl-umi3218/eigen-quadprog (accessed on 1 January 2023) to solve the QP problem for whole body trajectory tracking control and meet the control period requirement of 1 ms.

### 4.2. Arm Momentum Compensation Experiment

#### 4.2.1. Yaw Compensation Experiment

**Figure 4:**
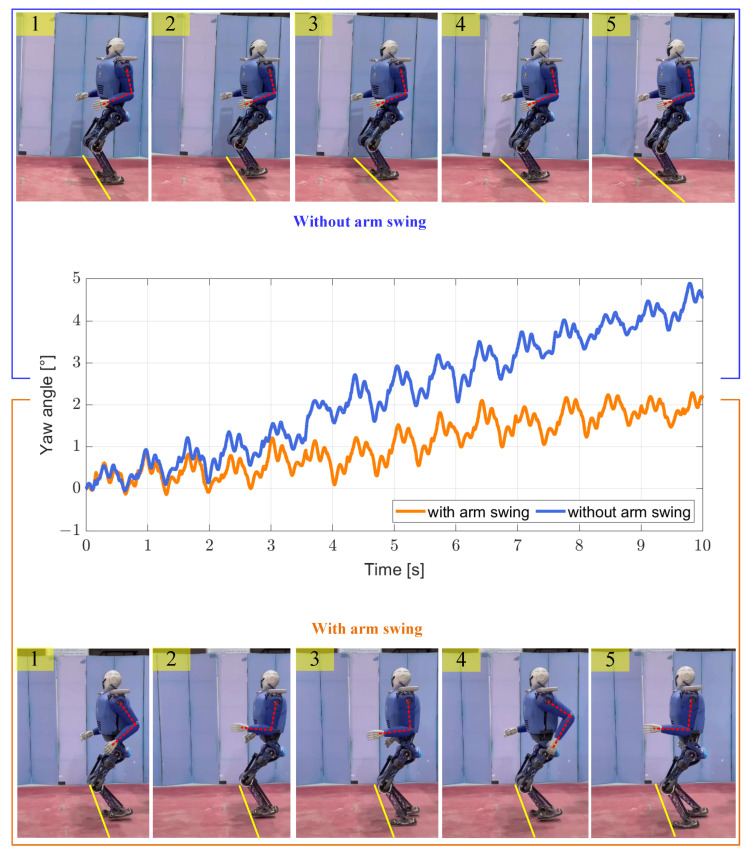
(**Top**) walking without arm swing movement. (**Middle**) yaw changes in the robot during walking without and with arm swing. (**Bottom**) walking with arm swing movement.

We let the robot walk in separate cases without an arm swing and with an arm swing. As shown in Figure 4 (top), when there is no arm swing, the yaw angle deviates after the robot walks in a straight line for a period of time. On the one hand, this deviation occurs because the friction moment between the supporting foot and the ground is smaller than the moment of inertia generated by the swing of the swinging leg that occurs during walking. On the other hand, when the robot walks dynamically, each adjustment is not fixed during the process of tracking the expected trajectory, which means that the switching processes of the left and right legs do not symmetrically offset the disturbances generated by each other.

As shown in Figure 4 (bottom), when there is an arm swing during the walking process, the deviation of the yaw angle during the walking process along a straight line is smaller than that without the arm swing. This occurs because the angular momentum generated by the swinging motion of the arm compensates to some extent for the adverse effects of the swinging leg. By performing this set of comparative experiments, we verified the inhibition effect of arm swings on the yaw direction drift in advance.

#### 4.2.2. Disturbed Equilibrium Restoration Experiment

**Figure 5:**
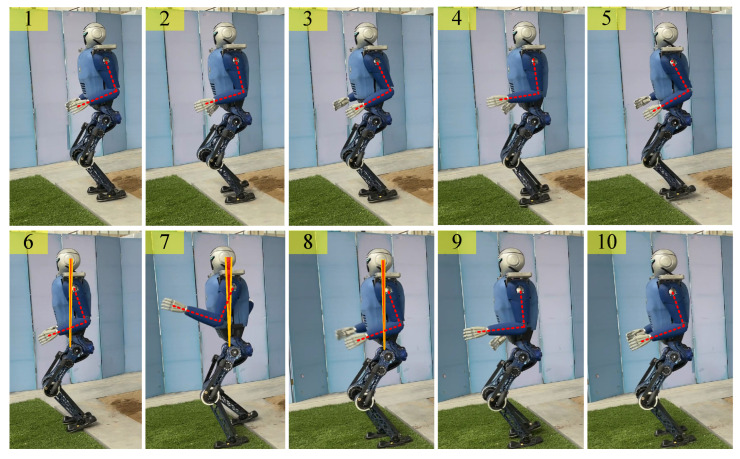
Video capture of the momentum compensation recovery of the swing arm when disturbed by the robot’s descending steps. The red fan-shaped area formed between the two yellow lines represents the upper body’s pitch range.

**Figure 6:**
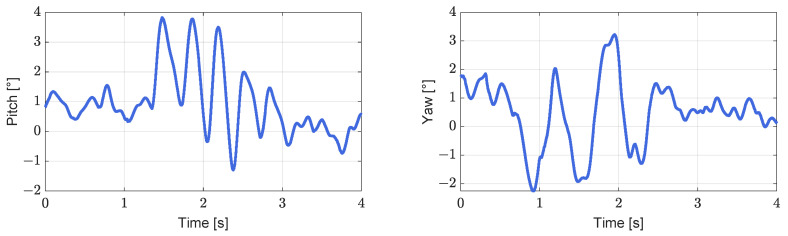
Changes in pitch (**left**) and yaw (**right**) angle during the downward step.

**Figure 7:**
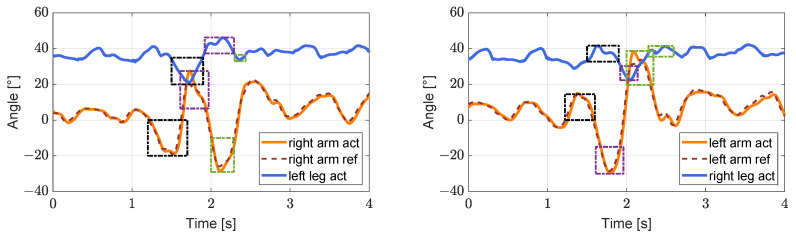
The images on the (**left**,**right**) show the reference angle (dark red dashed line), the actual angles (solid orange lines) of the right arm and the left arm shoulder, and the corresponding left and right leg hip joint pitch angles (solid blue lines), respectively. The black dash-dotted line box, the purple dash-dotted box, and the green dash-dotted box within the same color range indicate the associated changes.

**Figure 8:**
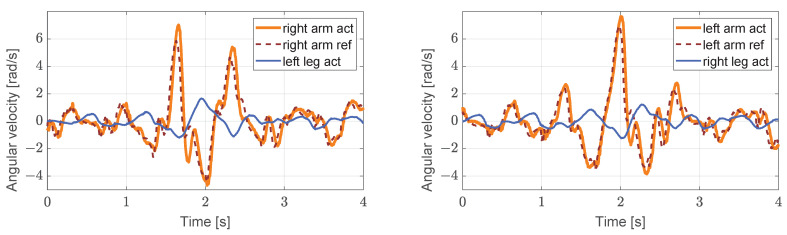
The images on the (**left**,**right**) show the reference angular velocity (dark red dashed lines), the actual angular velocities (solid orange lines) of the right arm and the left arm shoulder, and their corresponding left and right leg hip joint pitch angular velocities (solid blue lines), respectively.

We let the robot start on a platform and then walk from this platform, which is 5 cm high relative to the ground level (virtual grass), as shown in Figure 5. The robot’s arms can be seen to be swinging normally in the first to third screenshots. In the fourth screenshot, the left foot of the robot has partially stepped on the platform. Because of the slight disturbance caused, the swing amplitude of the arm increased slightly. In the seventh screenshot, there is a large fluctuation of the left foot, and a larger stride is taken. The upper body also leans forward, obviously, and the arm shows a corresponding very large swing range. The sixth to eighth screenshots illustrate the process of lowering the robot from the platform, and the red fan-shaped area in the figure clearly shows that the pitch angle of the upper body initially increases and then decreases, as shown in Figure 6 (left). Simultaneously, the yaw angle of the robot changes considerably during the process of descending the steps, but as a result of the action of the arm, the original walking direction is restored gradually, as shown in Figure 6 (right). From the results in Figure 7 and Figure 8, it is reasonable to deduce that part of the reason why the balance is restored, and straightness is maintained after the body is disturbed during this process is related to the positive effect of the arm swing.

As shown in Figure 7, the arm swing is synchronized substantially with the swing of the corresponding leg when the robot is not disturbed. However, when the robot walks off the platform, it is disturbed greatly. In addition, to compensate quickly for the effect of the disturbance, the arm swing is ahead of the leg swing, as shown in the black dash-dotted box, the purple dash-dotted box, and the green dash-dotted box in Figure 7. The forward swing of the arm will swing in the same direction as the ipsilateral leg at a specific moment (homolateral walking); this is caused by the rapid swinging of the arm to prevent the upper body from overturning. At this time, although such behavior will have an unfriendly enhancement effect on the offset in the yaw direction, it will also play a friendly role in avoiding instability.

In Figure 8, the arm and the leg swing at similar speeds without disturbance. However, when the robot stepped off the platform and was, thus, disturbed, the step length increased, and the swing angular velocities of the legs increased slightly, but the swing velocities of the arms changed dramatically. This behavior indicates that the arms are trying to compensate for the imbalance, and it also refers to the motion of the arm ahead of the leg indicated in Figure 7.

## 5. Conclusions

Based on the basic mechanism of human motion, the biped robot is designed to walk stably in a human environment, particularly after being disturbed, which represents the basic problem that affects its practical application. In this article, we make full use of the momentum compensation capability of the biped robot’s arms and propose a method of mixing linear momentum with angular momentum. An arm momentum compensation controller based on the dynamic state of the lower limbs causes the swinging motions of both arms to compensate for the disturbance caused by the stepping motions of both legs.

In the comparison experiment with and without arm swing performed on the BHR-B2 platform, the effectiveness of the arm compensation approach for the disturbance caused by the swinging leg on the contact between the supporting foot and the ground is verified by the difference of yaw angle during the linear walking of the robot, which greatly improves the yaw error of the robot. In the experiment where the robot descended steps, the robot did not establish knowledge of the terrain in advance. For the robot, the events that changed the expected walking state via leg swing were considered to be sudden and large disturbances. The arm swing was closely related to the change in the state of the lower limb. Therefore, after being disturbed, it was verified experimentally that the arm would swing greatly to compensate for such a sudden change, which allows the BHR-B2 to recover and stabilize quickly; in particular, the upper body recovered rapidly and became upright.

When the robot is walking on uneven ground, smooth plane and subjected to sudden external disturbance, the arm hybrid momentum compensation will greatly improve the walking stability and environmental adaptability. However, to satisfy the requirement for conservation of momentum, the swinging of the robot arm is symmetrical and, in most cases, is synchronized with the swinging of the leg on the opposite side. However, whether the asymmetric swinging of the arm and occasional asynchronous swinging with the leg on the opposite side will have a positive effect on a sudden large disturbance will form the subject of future study.

## Figures and Tables

**Table 1:** Structural parameters of the BHR-B2 robot.

Body Parts	Mass (kg)	Length (m)
Torso	29.3	0.60
Thigh	3.081	0.35
Calf	1.583	0.35
Arm	1.520	0.38 ^1^
Foot	0.635	0.19

^1^ We ignore the elbow joint of the arm, and, thus, the length of the arm is obtained using the theory of sines and cosines in the triangle formed by the upper and lower arms.

**Table 2:** Controller parameters of the BHR-B2 robot.

Name	Parameter
$\zeta_{a}$	0.8
$\zeta_{l}$	0.2
ζ	1.2
$k_{P_{1}}$	81
$k_{D_{1}}$	3.1
$k_{P_{2}}$	70
$k_{D_{2}}$	2.3
$\tau_{A}^{max}$	10 ^1^

^1^ The unit of torque is Nm.

## Data Availability

Not applicable.
